# Using binary optical elements (BOEs) to generate rectangular spots for illumination in micro flow cytometer

**DOI:** 10.1063/1.4963010

**Published:** 2016-09-28

**Authors:** Jingjing Zhao, Zheng You

**Affiliations:** 1 State Key Laboratory of Precision Measurement Technology and Instrument, Tsinghua University, Beijing 100084, China; 2 Department of Precision Instrument, Tsinghua University, Beijing 100084, China; 3 Beijing Laboratory for Biomedical Detection Technology and Instrument, Tsinghua University, Beijing 100084, China

## Abstract

This work introduces three rectangular quasi-flat-top spots, which are provided by binary optical elements (BOEs) and utilized for the illumination in a microflow cytometer. The three spots contain, respectively, one, two, and three rectangles (R1, R2, and R3). To test the performance of this mechanism, a microflow cytometer is established by integrating the BOEs and a three-dimensional hydrodynamic focusing chip. Through the experiments of detecting fluorescence microbeads, the three spots present good fluorescence coefficients of variation in comparison with those derived from commercial instruments. Benefiting from a high spatial resolution, when using R1 spot, the micro flow cytometer can perform a throughput as high as 20 000 events per second (eps). Illuminated by R2 or R3 spot, one bead emits fluorescence twice or thrice, thus the velocity can be measured in real time. Besides, the R3 spot provides a long-time exposure, which is conducive to improving fluorescence intensity and the measurement stability. In brief, using the spots shaped and homogenized by BOEs for illumination can increase the performance and the functionality of a micro flow cytometer.

## I. INTRODUCTION

Flow cytometers are powerful instruments to analyze bio particles in flows.^1,2^ Because of the success of conventional flow cytometers and the advances in microfluidics, micro flow cytometers have gained much attention of researchers^3,4^ and have been widely used for bio-analysis.^5–8^ For micro flow cytometers, there are two fundamental techniques: laser spot for illumination and microfluidic chip for sample flow focusing. Generally, elliptical focal spots of 5–20 *μ*m high and 100 *μ*m wide are applied in the conventional flow cytometers, which can be produced by two crossed cylindrical lenses of different focal lengths.^2^ For micro flow cytometers, circular spots and elliptical spots are both applied. The former can be obtained by using a high power objective to focus on a laser beam,^9–11^ and the latter can be achieved through combining an object with a cylindrical lens^12–14^ or a slit^15–17^ to shape a laser beam. Besides, some efforts have been made to integrate optical illumination into microfluidic chips, including using optical waveguides to deliver laser^18–20^ and planar lenses patterned into or onto the microchannel walls to focus on laser.^21–24^ However, these on-chip elements cannot provide a focused spot as small as the off-chip lenses do. Apart from geometrical optics based on reflection and refraction, diffractive optical elements have been used to generate line-shaped spots with small size,^25^ introducing diffraction into microflow cytometry. All of the above spots feature Gaussian intensity distributions. Their illuminations are not uniform and boundaries are not straight or sharp, which means the fluorescence emitted from a cell or a particle will be affected by its route passing through the spot, including duration time, intensity, and waveform. Therefore, the ideal spots should be flat-top and rectangular. In fact, some commercial flow cytometers start using the rectangular spots,^26^ which are generated by several prisms and optical lens based. In this work, a binary optical element (BOE) with a lens is proposed to generate a quasi flat-top and rectangular beam spot. As a diffractive optical element, BOE is good at beam shaping and homogenization.^27^ In this paper, three BOEs are designed, giving three rectangular quasi-flat-top spots. The first spot is a 50 *μ*m × 10 *μ*m rectangle (R1 spot), the second one contains two rectangles (R2 spot), and the third is composed of two rectangles and a 50 *μ*m square (R3 spot). Each of the three spots has its own advantages and will be discussed in detail in Section II A.

The other crucial technique of a microflow cytometer is the microfluidic chip,^28–34^ which is used for squeezing the sample flow both in horizontal and vertical directions and making it into a narrow stream along the channel centerline. In the narrow stream, it is used to make the particles contained in the sample flow have almost identical flow and illumination conditions, which is necessary for measurement accuracy and stability. Based on our previous work,^35^ a microfluidic chip is developed for 3D hydrodynamic focusing. This chip has six curved channels for sheath flows and one straight channel for the sample flow, and implements the vertical and horizontal focusing of sample flow successively. In order to simplify fluid control, the channel flow resistances are specially designed according to the relationship between the flow resistances and the flow rates of channels. So that the sheath-flow channels can be connected to the same fluid source, and only one sheath flow and one sample flow need manipulating. Demonstrated by experiments, the chip is able to accomplish 3D focusing with the comparable performance as conventional flow cytometers do, in terms of flow velocity (0.7–9.0 m/s), sample flow rate (10–120 *μ*l/min), and cross-sectional dimensions of the focused sample flow (10–23 *μ*m). A micro flow cytometer is built using the BOEs and the focusing chip. Fluorescence microbeads are detected, good coefficients of variation (CVs) are achieved, and the flow velocity can be measured.

The major significance in this work is presenting the possibility of utilizing BOEs to generate spots of special shapes and desired intensity distributions for the applications in microflow cytometers, and potentially for other microfluidic systems.

## II. DEVICES AND SYSTEM

### A. BOEs for rectangular spots

**FIG. 1.**
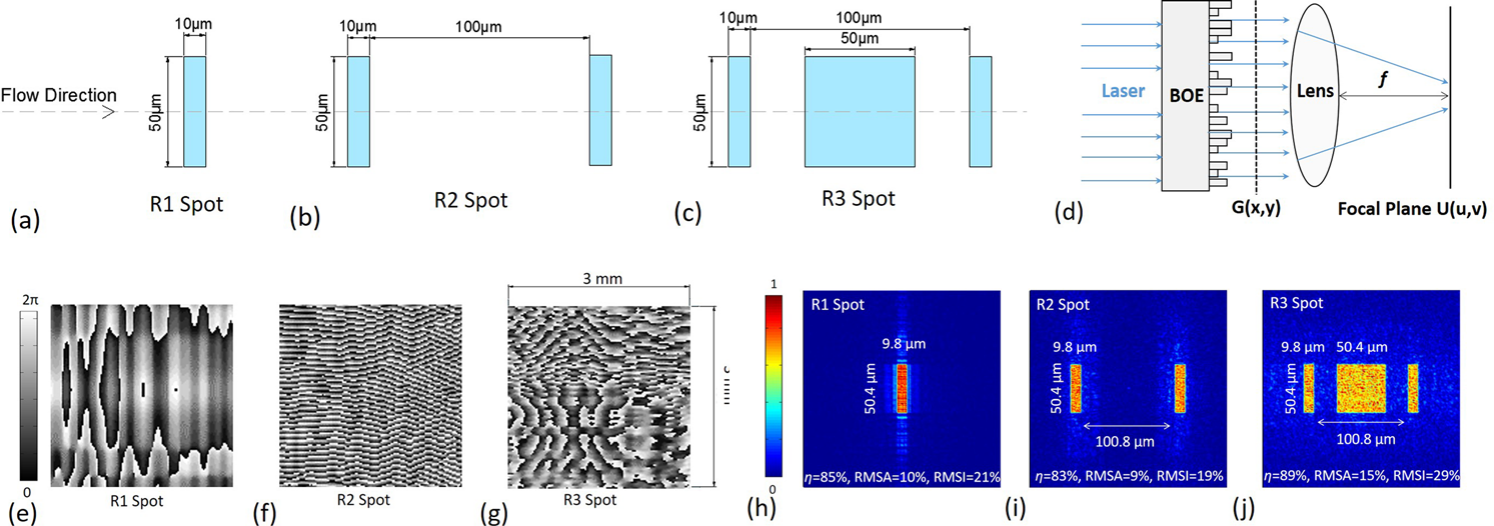
Three rectangular spots: (a) R1 spot, (b) R2 spot, and (c) R3 spot; (d) the BOE optical system; the discretized solutions of the phase modification Φ_B_(*x*,*y*) for the three spots: (e) R1 spot, (f) R2 spot, and (g) R3 spot; the focal spots given by the discretized solutions: (h) R1 spot, (i) R2 spot, and (j) R3 spot.

Three rectangular spots with flat tops are proposed in Figs. 1(a)–1(c), containing, respectively, one, two, and three rectangles, of which the long sides are perpendicular to the flow direction. R1 spot is an improvement for the conventional elliptical Gaussian spot. Compared with R2 and R3 spots, R1 is the smallest in size, leading to a higher spatial resolution and throughput, as well as larger light intensity, signal amplitude, and signal-to-noise ratio (SNR). R2 and R3 spots comprise more than one pattern, thus one bead passing through R2 or R3 spot emits fluorescence twice or thrice, and thus the flow velocity can be measured in real time. Researchers^36^ have confirmed that increasing the time exposure to the laser spot can improve the fluorescence intensity and the measurement stability. For this purpose, R3 spot is designed with a long exposure time, by adding the square in the middle compared with the R2 spot.

To implement the three spots, BOEs are used, which are planar elements with a multi-level surface relief structure. In this structure, the phase of the incident waves is modified by the geometry of the surface relief pattern, and the desired focal spots can be formed by the interference of the waves emitted from the different zones on the surface. The optical system for BOE is shown in Fig. 1(d). The incident laser *G_0_*(*x*,*y*) is *A*(*x*,*y*)exp[iΦ_0_(*x*,*y*)], where *A*(*x*,*y*) is the amplitude and Φ_0_(*x*,*y*) is the phase of the light field. TEM_00_ mode laser is utilized, thus Φ_0_(*x*,*y*) is constant. Passing through the BOE, the laser *G*(*x*,*y*) is *A*(*x,y*)exp[iΦ_*0*_(*x,y*) + iΦ_*B*_(*x,y*)], where Φ_*B*_(*x,y*) is the phase modification introduced by BOE. *U*(*u*,*v*) represents the field in the focal plane, of which the relationship with *G*(*x*,*y*) is given below

$$\left\{ \begin{array}{c} U\left(u,v\right)=\frac{1}{\lambda f}\iint G\left(x,y\right)\text{exp}\left[-i2\pi \left(xf_{x}+yf_{y}\right)\right]dxdy=\frac{1}{\lambda f}\text{FT}\left[G\left(x,y\right)\right]\\ G\left(x,y\right)=A\left(x,y\right)\text{exp}\left[i\Phi_{0}\left(x,y\right)+i\Phi_{B}\left(x,y\right)\right]\\ \left(f_{x},f_{y}\right)=(\frac{u}{\lambda f},\frac{v}{\lambda f}), \end{array}\right.$$ (1)

where (*f_x_*, *f_y_*) represents the spatial frequency, *f* is the lens focal length, *λ* is the laser wavelength, and FT means Fourier transformation. In this design, the BOE is a square of a 3 mm side, and there are 128 × 128 square zones of a 23.4 *μ*m side on the BOE surface. The focal length is 10 mm, the laser is 488 nm with a diameter of 3 mm, and the sampling interval on the focal plane is given by *λf*/*D* = 1.63 *μ*m. Obviously, the key to generate a desired spot is to find out the solution of Φ_*B*_(*x,y*). It is given by a modified Gerchberg–Saxton (GS) algorithm,^37,38^ which is an iterative method based on the Fourier transform pair of *G*(*x*,*y*) and *U*(*u*,*v*). Besides, the relationship between Φ_B_(x,y) and the surface relief pattern *h*(*x*,*y*) is

$$h\left(x,y\right)=\frac{\lambda }{2\pi \left(n-1\right)}\Phi_{B}\left(x,y\right),$$ (2)

where *n* is the BOE refractive index and *h*(*x*,*y*) is the relief height. Generally, BOEs are fabricated through photolithography and etching processes. When using *N* masks, 2^*N*^ surfaces are produced and the height interval is *λ*/[2^*N*^ (n–1)]. Accordingly, Φ_*B*_(*x,y*) needs discretizing to 2^*N*^ phase levels with the interval of π/2^*N*–1^. Here, *N* is 4. The discretized solutions of Φ_*B*_(*x,y*) for the three spots are shown in Figs. 1(e)–1(g) with the focal spots illustrated in Figs. 1(h)–1(j). Three parameters are used for evaluation: the diffractive efficiency *η*, the relative root mean square errors of amplitude and intensity (RMSA and RMSI) of a spot

$$\eta =\frac{\iint U_{S}{\left(u,v\right)}^{2}dudv}{\iint A{\left(u,v\right)}^{2}dxdy},$$ (3)

$$\text{RMSA}=\frac{\sqrt{\iint {\left[\left|U_{S}\left(u,v\right)\right|-\text{mean}\left(\left|U_{S}\left(u,v\right)\right|\right)\right]}^{2}dudv}}{\text{mean}\left(\left|U_{S}\left(u,v\right)\right|\right)},$$ (4)

$$\text{RMSI}=\frac{\sqrt{\iint {\left[U_{S}{\left(u,v\right)}^{2}-\text{mean}\left(U_{S}{\left(u,v\right)}^{2}\right)\right]}^{2}dudv}}{\text{mean}\left(U_{S}{\left(u,v\right)}^{2}\right)},$$ (5)

where *U_S_*(*u*,*v*) refers to the spot region. The spots in Figs. 1(h)–1(j) are not absolutely uniform, as reflected by RMASs and RMSIs, which is a common limitation of BOE homogenizers. However, the dimensions are very close to the desired values with errors of 1%–2%, and boundaries are pretty sharp and straight.

**FIG. 2.**
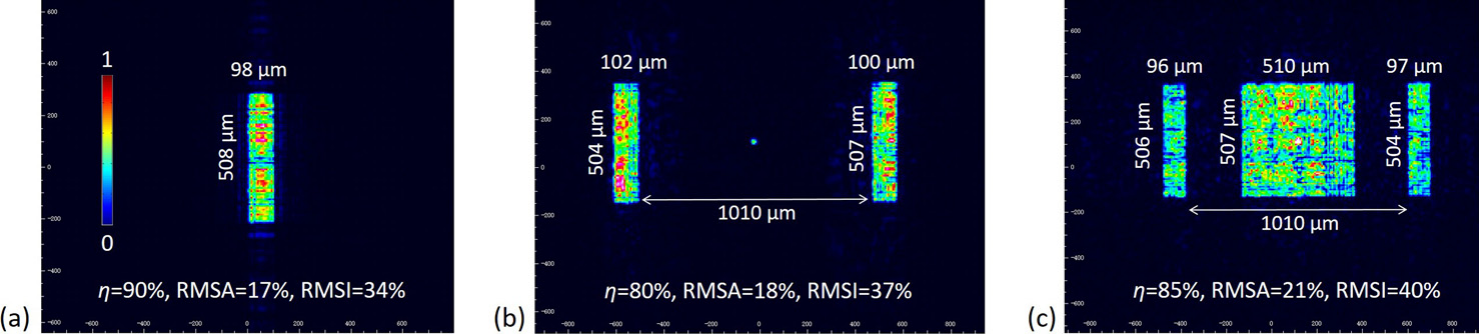
The intensity distributions of the three BOEs captured by the profiler, and the spots at ten-fold magnification: (a) R1 spot, (b) R2 spot, and (c) R3 spot. The dimensions were measured by imaging processing in Matlab.

Finally, the three designs were fabricated on quartz plates (*n* = 1.4632 at 488 nm) with a 66 nm height interval and were tested using a power meter (Coherent, FieldMaxII) and a laser beam profiler (Thorlabs, BC106N-VIS). Referring to the BOE illumination system in Fig. 1(d), the optical sensor of the meter or profiler was set at the lens focal plane. Taking into account that the pixel size of the profiler is 6.45 *μ*m, a lens with a 100 mm focal length was used instead of the 10 mm focal length in order to improve measurement precision (the 10 mm focal length was used when running the micro flow cytometer), and the spot dimensions were magnified tenfold. The experimental results are shown in Fig. 2. Compared with the designs, the real spots still own high efficiencies, accurate dimensions, and sharp boundaries, but the uniformities decrease because of the fabrication errors. One way to increase the uniformity is to enhance the BOE size and the incident laser diameter, since the sampling interval (=*λf*/*D*) on the focal plane is in inverse proportion to the BOE side length *D* (equals to the laser diameter). In sum, the fabricated BOEs provided desired rectangular spots with quasi-flat tops.

### B. Hydrodynamic focusing chip

**FIG. 3.**
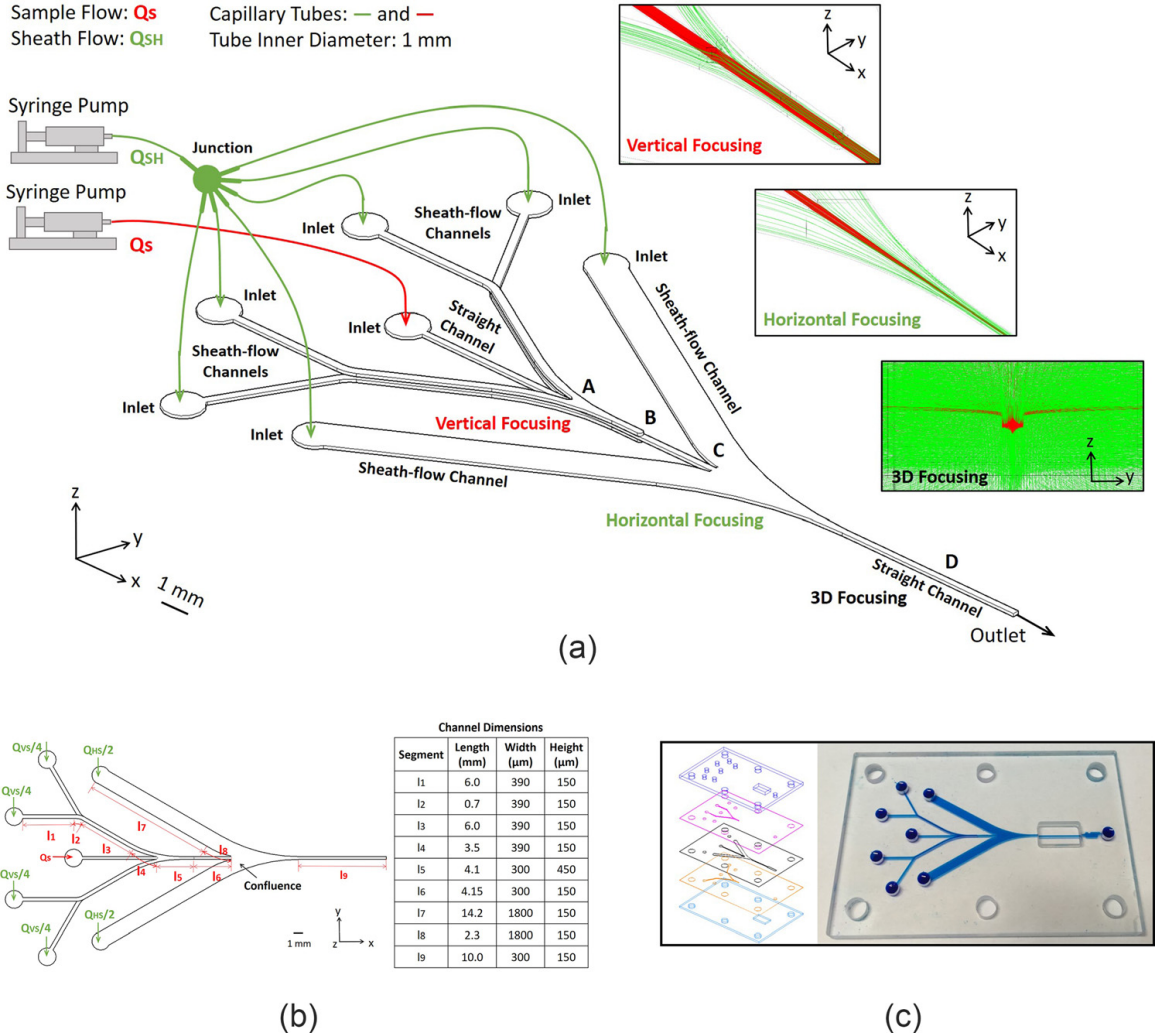
(a) Schematic diagram of the focusing chip, (b) dimensions of the microchannels, and (c) the chip photograph.

The focusing chip is illustrated in Fig. 3(a). The symmetric structure comprises three 150 *μ*m thick layers of rectangular channels, including six channels for the sheath flow and one straight channel for the sample flow. The sample flow is first confined vertically by four sheath flows from above and below, and then squeezed horizontally by two flows from the right and left sides. To keep flows laminar and stable, especially at high velocities of several meters per second, the profiles of the channels are modeled and optimized, and the design processes are presented in our work.^35^ Every sheath-flow channel contains a curved segment at the connection to the straight channel. Thus, the sheath flows can be gradually guided to the direction parallel to the sample flow and smoothly stream into the straight channel, keeping and focusing the process stable.

In operation, one syringe pump is controlled to supply the sample slow and another pump for the sheath flow. The pumps are connected to the inlets of the microchannels using the off-chip capillary tubes of 1 mm inside diameter. The sheath flow *Q_SH_* is divided into six subflows, and the flow for vertical focusing is *Q_VS_* and the flow for horizontal focusing is *Q_HS_* (*Q_SH_* = *Q_VS_* + *Q_HS_*), as shown in Fig. 3(b). Since the tube cross section is much larger than those of the sheath-flow channels, the pressure drops in the off-chip tubes can be ignored when compared with the drops in the microchannels. Approximately, the sheath-flow channels have the same inlet pressures and the same pressure drops between their inlets and the confluence. According to the above analysis and hydrodynamic principles, two pressure drops, Δ*P*_1_ and Δ*P*_2_, can be formulated below

$$\begin{array}{c} \Delta P_{1}=\sum_{i=1}^{6}R_{i}Q_{i}=\sum_{i=1}^{6}\frac{32\upsilon \rho l_{i}}{d_{i}^{2}}V_{i}=\sum_{i=1}^{6}\frac{32\upsilon \rho l_{i}}{d_{i}^{2}}\left(\frac{Q_{i}}{A_{i}}\right)=32\upsilon \rho \left[\frac{l_{1}+l_{2}+l_{3}+l_{4}}{d_{1}^{2}}\left(\frac{Q_{VS}/4}{A_{1}}\right)+\frac{l_{5}}{d_{5}^{2}}\left(\frac{Q_{VS}+Q_{S}}{A_{5}}\right)+\frac{l_{6}}{d_{6}^{2}}\left(\frac{Q_{VS}+Q_{S}}{A_{6}}\right)\right]\\ \approx 32\upsilon \rho \left[\frac{l_{1}+l_{2}+l_{3}+l_{4}}{d_{1}^{2}}\left(\frac{Q_{VS}/4}{A_{1}}\right)+\frac{l_{5}}{d_{5}^{2}}\left(\frac{Q_{VS}}{A_{5}}\right)+\frac{l_{6}}{d_{6}^{2}}\left(\frac{Q_{VS}}{A_{6}}\right)\right], \end{array}$$ (6)

$$\Delta P_{2}=32\upsilon \rho K_{P}\left[\frac{l_{7}+l_{8}}{d_{7}^{2}}\left(\frac{Q_{HS}/2}{A_{7}}\right)\right],$$ (7)

$$d_{i}=\frac{2a_{i}b_{i}}{a_{i}+b_{i}},$$ (8)

$$\Delta P_{1}=\Delta P_{2},$$ (9)

where *R* is the flow resistance, *V* is the average flow velocity, *υ* is the kinematic viscosity of the fluid, *ρ* is the fluid density, *Q* is the flow rate, *a* is the channel width, *b* is the channel height, *l* is the channel length, *A* is the channel cross-sectional area, *d* is the hydraulic diameter, *K_P_* is the compensating factor for the high aspect-ratio channel, and subscript represents different channel segments. The rate ratio of *Q_HS_* to *Q_VS_* can be given by

$$\frac{Q_{HS}}{Q_{VS}}=\left(\frac{l_{1}+l_{2}+l_{3}+l_{4}}{4A_{1}d_{1}^{2}}+\frac{l_{5}}{A_{5}d_{5}^{2}}+\frac{l_{6}}{A_{6}d_{6}^{2}}\right)/\left(\frac{l_{7}+l_{8}}{2A_{7}d_{7}^{2}}K_{P}\right)+K_{Q},$$ (10)

where *K_Q_* is another compensating factor. Determined from simulations using the COMSOL software, the values of *K_P_* and *K_Q_* are 1.4 and 0.75, respectively. It shows that the desired ratio can be realized by adjusting the channel lengths. From simulations, the optimal value of the ratio of *Q_HS_/Q_VS_* is 8. In this situation, the focused sample flow has a quasi-square cross section, of which the height and the width are close to each other. Besides, velocity profile in a rectangular channel can be given by^39^

$$v=\frac{4b^{2}}{\mu \pi^{3}}\left(-\frac{dP}{dx}\right)\sum_{n=0}^{\infty }\frac{{\left(-1\right)}^{n}}{{\left(2n+1\right)}^{3}}\left\{1-\frac{\cosh\left[\left(2n+1\right)\pi y/b\right]}{\cosh\left[\left(2n+1\right)\pi a/2b\right]}\right\}\;\text{cos}\;\frac{\left(2n+1\right)\pi z}{b},$$ (11)

where *P* is the pressure and *μ* is the dynamic viscosity. The maximum velocity *V*_MAX_ is at the center, representing the velocity of the focused sample flow, and equals 2.05 times the average velocity *V*_AVR_ in this design.

The chip was constructed by integrating five glass plates via UV adhesive bonding, with a photo shown in Fig. 3(c). A 1 mm plate and a 2 mm plate served as covers to protect the inner structure and offered the inlets and outlets. The microchannels were carved by precision machining. Due to fabrication errors generated during grinder processing, the cross-sectional dimensions of the straight channel are 310 *μ*m × 163 *μ*m and the designed values are 300 *μ*m × 150 *μ*m. The focusing performances of the chip were experimentally characterized and analyzed in the Appendix.

### C. Micro flow cytometer

**FIG. 4.**
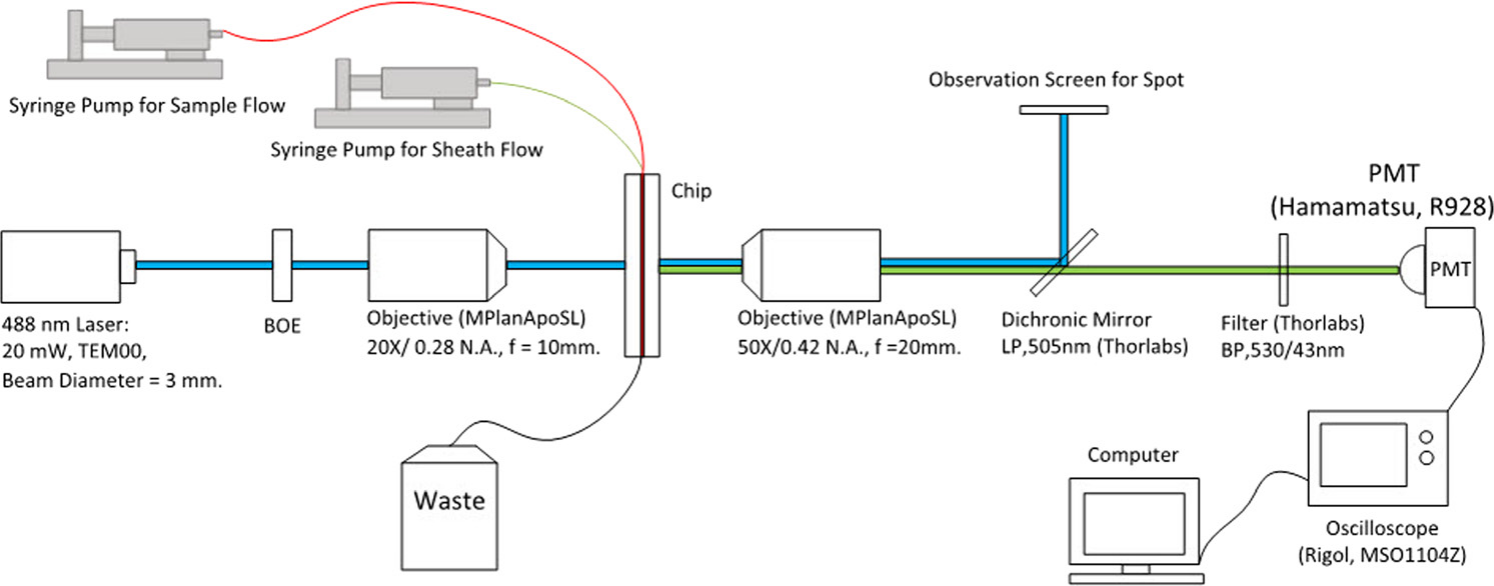
The micro flow cytometer system.

The micro flow cytometer is established on the basis of the focusing chip and the BOEs, as shown in Fig. 4. Fluorescence microbeads (Huge Biotech, Inc, Polystyrene Spheres, 488 nm/530 nm) of 5 *μ*m diameters are suspended in a distilled water with a concentration of 5 × 10^6^ particles/ml, serving as the sample fluid, and the sheath fluid was distilled water. The sample flow and the sheath flow are powered by two syringe pumps (LongerPump, Inc., TS-1B). The incident laser is shaped and homogenized by the BOE and further focused by the objective, forming the desired spot at the focal plane. Beads are excited by the spot and emit fluorescence, which is gathered by the other objective and detected by a photomultiplier tube (PMT, Hamamatsu, R928 + C7427). The PMT signals are displayed on an oscilloscope (Rigol, MSO1104Z, Sampling rate = 1 GSa/s) and further recorded and analyzed in a computer. The distance between the objective and the chip is adjusted to make the focused sample flow at the focal plane. If not, beads will be illuminated by a deformed spot, which can influence the fluorescence intensity, as presented in Figs. S1–S3 in the supplementary material. The three spots are, respectively, tested. The laser power is 20 mW constantly. As discussed in Section II A, the intensity of R1 spot is the highest, R2 intensity is medium, and R3 intensity is the lowest. In order to increase the signal amplitude and SNR, the PMT gain is increased when using R2 or R3 spot, and average filtering is performed.

## III. RESULTS AND DISCUSSION

### A. Fluorescence signal

**FIG. 5.**
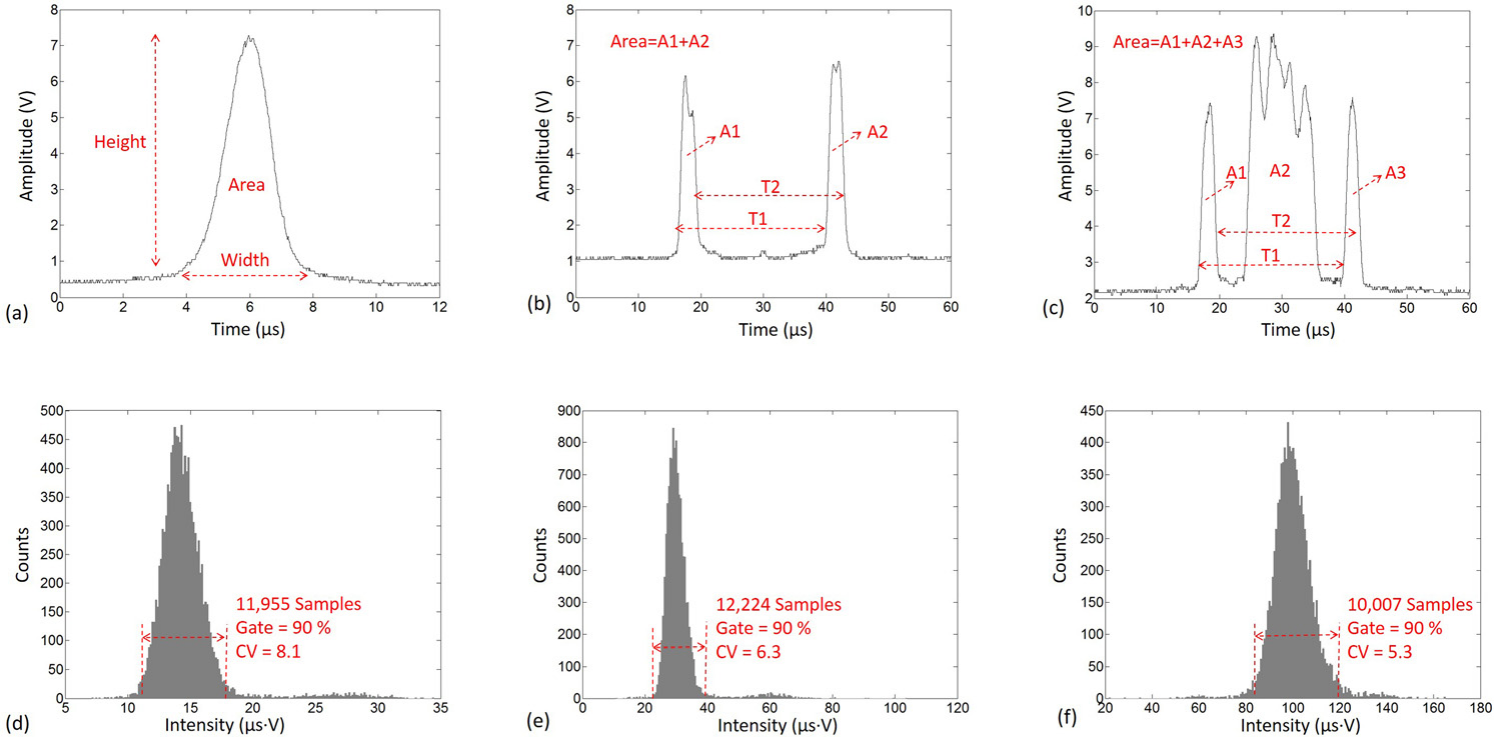
The fluorescence signals when using (a) R1 spot, (b) R2 spot, and (c) R3 spot for illumination. When using R2 spot and R3 spot, the area is the sum of the areas of the two/three pluses. The intensity histograms for (d) R1 spot, (e) R2 spot, and (f) R3 spot.

The micro flow cytometer was operated at the sample flow rate of 60 *μ*l/min and the sheath flow rate of 7.2 ml/min, and the throughput was more than 4000 eps. The fluorescence signals of a bead illuminated by the three spots are shown in Figs. 5(a)–5(c) and Video S1 in the supplementary material. Due to the quasi-flat-top spots, the waveform tops were also quasi-flat, especially for those excited by R2 or R3 spots. While these waveforms had almost identical characteristics, including the fluctuations, as shown in Fig. S4 in the supplementary material. The height, width, and area of a signal were measured, and the area was used to represent the fluorescence intensity. For R2 and R3 spots, the area was the sum of the areas of the two/three pluses. The common index to evaluate the measurement stability for flow cytometers was coefficient of variation (CV). As shown in Figs. 5(d)–5(f), there were three intensity histograms of beads, with a 90-percent-gate to exclude the events seriously deviating from the mean, and the CV of the events inside the gate was utilized for evaluation. For credibility, more than 10 000 events were collected for every CV in this work. Obviously, the signal width of R1 spot was much shorter than R2 and R3 spots, thus higher throughput could be achieved. We had tested a sample fluid of 2.5 × 10^7^ particles/ml with R1 spot, and the throughput reached as high as 20 000 eps. When using R2 or R3 spot, the bead velocity could be extracted from the signal, as shown in Figs. 5(b) and 5(c). The velocity was measured, respectively, with the separation between the rising edges of two rectangular sub-spots and the separation between the falling edges, and the average value was used to reduce the measuring errors. It was calculated as (*L*/*T*_1_ + *L*/*T*_2_)/2, where *T*_1_ and *T*_2_ were the time intervals and *L* was the separation, of which the value was 110 *μ*m according to the spot dimensions in Fig. 1.

### B. CVs of fluorescence microbeads

**FIG. 6.**
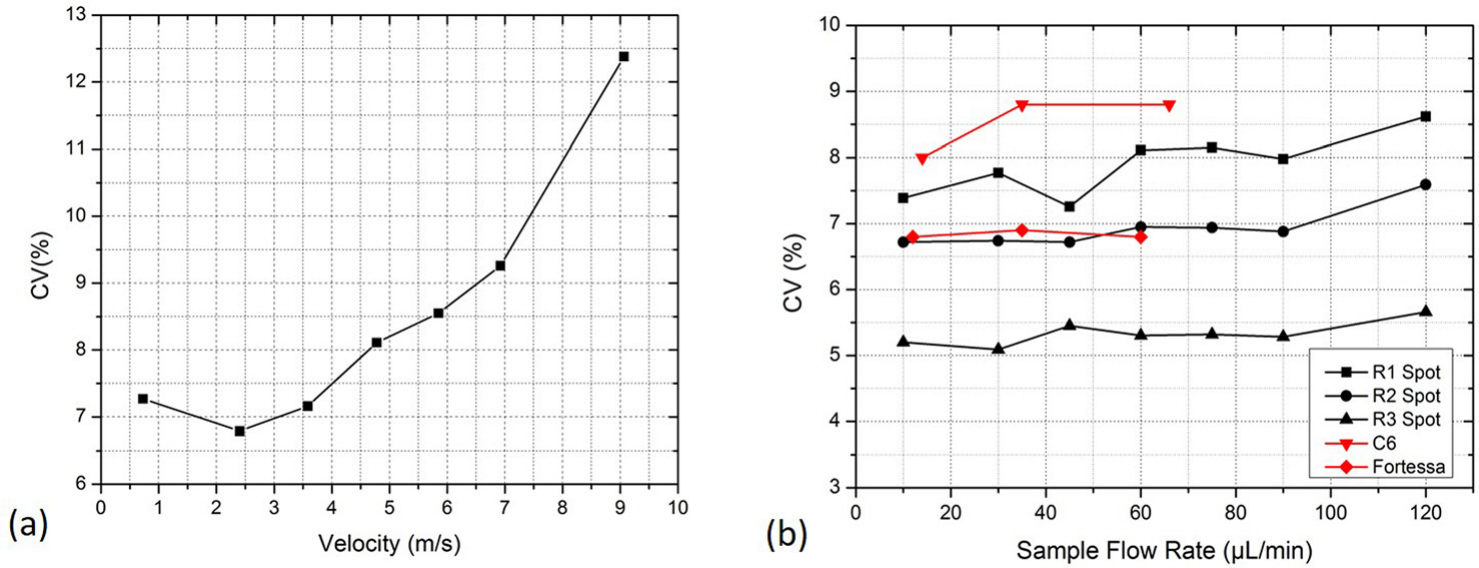
(a) The experimental CVs with the sample flow velocity from 0.7 to 9.0 m/s. (b) The experimental CVs of R1–R3 spots when the microflow cytometer operated at 4.8 m/s and the sample flow rate increased from 10 to 120 *μ*l/min, and the CVs given by two commercial instruments, BD LSR Fortessa and BD Accuri C6.

**FIG. 8.**
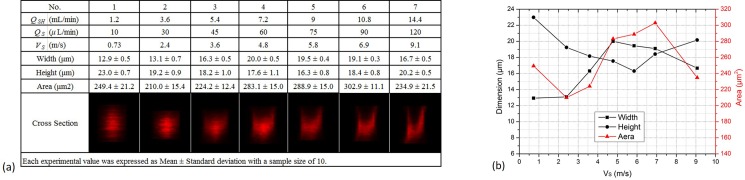
Experimental results of the focused sample flow when the sample flow velocity increased from 0.7 to 9.1 m/s, including (a) experimental conditions and the cross sections, (b) height, width, and area.

Two experiments were performed on the microflow cytometer, and the flow rates were in accordance with those of the experiments in the Appendix. In the first experiment, beads were illuminated by the R1 spot and tested in the velocity range of 0.7–9.0 m/s. The CV increased with the velocity, as shown in Fig. 6(a), indicating that the measurement stability decreased at high velocities. There were two reasons. First, at low velocities, the beads in the sample flow achieved a long-time exposure to a laser spot and emitted stronger fluorescence, increasing accuracy and stability. Second, as presented in Fig. 8 of the Appendix, the cross section of the sample flow became scattered at high velocities, thus it expanded the distribution of beads in the cross section and further enlarged the illumination differences among beads, resulting in the decline in stability. In summary, high velocities brought with high throughput and low velocities gave high performance. In practice, a commercial flow cytometer operates under the condition that the sample flow rate changes but the velocity remains constant. Considering that the commercial flow cytometers work at 3–6 m/s, the operating velocity for the system is set at 4.8 m/s, as a result of the compromise between efficiency and performance.

In the second experiment, the sample flow rate varied between 10 and 120 *μ*l/min at the velocity of 4.8 m/s. R1, R2, and R3 spots were tested with results in Fig. 6(b). The CV increased with the sample flow rate, which could be explained by the expansion of the cross section of the sample flow. The CVs derived from the R3 spot were the lowest, the CVs from the R2 spot were medium, and those from the R1 spot were the highest. It experimentally demonstrated that increasing the exposure time (or large illumination area) is an effective approach to improving the measurement stability. From Video S1, we can see that the amplitude fluctuations of the signals when using the R3 spot were much smaller than those of the R1 and R2 spots. Besides, when using R2 or R3 spot for illumination, CV changed little under 90 *μ*l/min, meaning that the two spots could offer stable performances over a wide rate range. The sample fluids were also analyzed in two commercial instruments, BD LSR Fortessa and BD Accuri C6 (using elliptical Gaussian spot), both of which ran at low, medium, and high rates. All the three spots provided smaller CVs in comparison with C6. When compared with Fortessa, the R1 spot was inferior, the R2 spot was equal, and the R3 spot was obviously superior. These spots given by BOEs presented excellent performances comparable with commercial cytometers, which are hard to achieve in microflow cytometers.^5,25,30,40^ In addition, the micro flow cytometer with a circular Gaussian spot was also tested (the system in Fig. 4 without using the BOEs), but the yielded CVs were more than 20%, further demonstrating the improvements achieved by the BOEs.

### C. Velocity measurement

Signals from R2 and R3 spots offered the velocity information. According to the experiment in the Appendix, the sample velocity was controlled from 0.7 to 9.0 m/s. By the measurement method proposed above, the velocities were calculated. For references, the velocities were predicted in theory using Equation (11) and simulated using COMSOL. All the results are listed in Table I. The experimental velocities were pretty close to the simulated and theoretical velocities, showing that the measurement method was feasible and accurate. The velocity CVs were small, demonstrating that the flows in the microchannel were stable and the sample flow with the beads was focused at the channel center.

**TABLE I.** Sample flow velocities.

		R2 spot		R3 spot	
Theoretical velocity (m/s)	Simulated velocity (m/s)	Velocity (m/s)	Velocity CV (%)	Velocity (m/s)	Velocity CV (%)
0.79	0.79	0.73	3.8	0.73	2.2
2.37	2.28	2.40	1.4	2.40	1.2
3.56	3.45	3.58	1.1	3.58	1.4
4.75	4.61	4.69	1.8	4.73	1.3
5.94	5.78	5.86	2.5	5.80	1.3
7.12	6.94	6.89	2.6	6.86	2.8
9.50	9.26	8.74	5.3	8.98	3.0

## IV. CONCLUSIONS

This work demonstrates using BOE to generate rectangular quasi-flat-top spots for a microflow cytometer. A microfluidic chip is also developed, whose 3D hydrodynamic focusing ability is comparable to a commercial flow cytometer. Thanks to the specially designed channel flow resistances, the chip is easy to operate since only two flows are needed, one for sample flow and the other for sheath flow. By integrating the BOEs and the chip, a microflow cytometer is established. CVs of fluorescence beads counted in the microflow cytometer are close to or even better than those derived from commercial instruments. Moreover, bead velocity can be measured in real time. The uniformities of the spots can be further improved by increasing the BOE size and the laser diameter. The major significance in this work presents the possibility of utilizing the specially designed BOEs to generate desired spots to meet different measurement demands and improve the performance in microflow cytometers, or even other microfluidic systems.

## V. SUPPLEMENTARY MATERIAL

See supplementary material for Video S1 that shows the fluorescence signals of a bead illuminated by the three spots. Figures S1–S3 show the deformed spots at the planes before or behind the focal plane and the fluorescence signals. Figure S4 contains five randomly selected fluorescence signals by the R3 spot.

## Figures and Tables

**FIG. 7.**
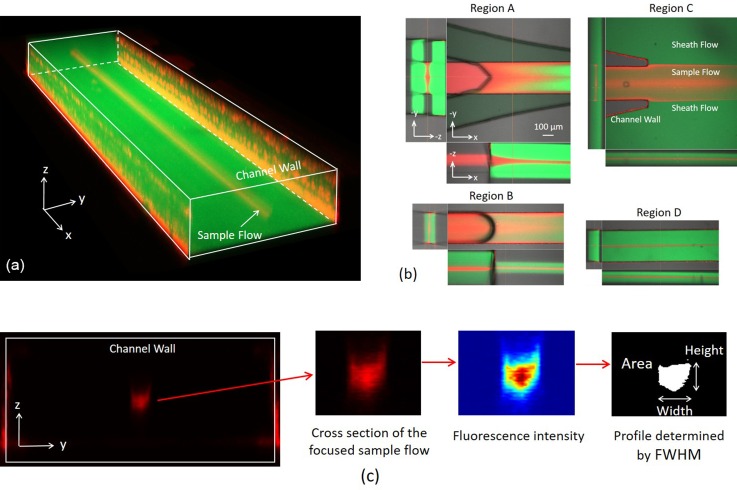
Confocal images of the 3D focusing. (a) The focused sample flow was a narrow stream along the centerline in the straight channel. (b) Flow configurations in four key regions, and the regions are indicated in Fig. 3(a). (c) Using image processing to quantify the cross-sectional dimensions of the focused sample flow.

**FIG. 9.**
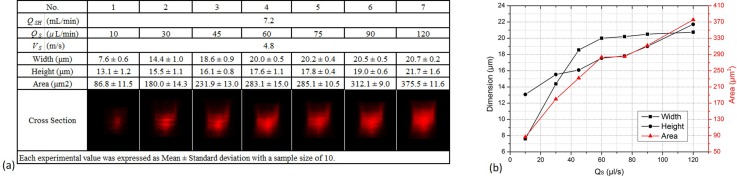
Experimental results of the focused sample flow when the chip operated at a constant velocity of 4.8 m/s and the sample flow rate increased from 10 to 120 *μ*l/min, as shown in (a) the table and (b) the figure.

## APPENDIX: HYDRODYNAMIC FOCUSING PERFORMANCES

To visualize the focusing behaviors of the microfluidic chip, confocal imaging was carried out by Nikon A1, using a rhodamine B aqueous solution (red) as the sample fluids and a sodium fluorescein aqueous solution (green) as the sheath fluids. Fig. 7(a) demonstrates that 3D hydrodynamic focusing was achieved in the straight channel. The confocal images of the key regions (regions A–D indicated in Fig. 3(a)) are shown in Fig. 7(b). Vertical focusing started in region A and ended in region B. Owing to the above and below sheath fluids, the sample flow became a thin layer and was exactly positioned in the vertical center plane of the channel. Next, in region C, the sample flow began to be pressed by the sheath fluids from left and right sides and the pressing strength gradually increased until the sample flow entered the following straight channel, where 3D focusing was complete and now the sample flow was a narrow stream at the channel center, as shown in region D. The dimensions of the cross section of the focused sample flow were measured, with the cross section profile determined by the full width at half-maximum (FWHM) of the fluorescence intensity distribution,^41,42^ giving height, width, and area, as shown in Fig. 7(c).

The focusing performance of the chip was characterized via two experiments. In the first experiment, the chip was demonstrated to have the capability of achieving 3D focusing in a wide velocity range. The flow rates *Q_SH_* and *Q_S_* were *k* × 120 *μ*l/min and *k* × 7.2 ml/min. The values of *k* were 1/6, 0.5, 0.75, 1, 1.25, 1.5, and 2, resulting in the sample flow velocity *V_S_* varying between 0.7 and 9.1 m/s. The results are shown in Fig. 8. When increasing the velocity, the cross-sectional profiles obtained from confocal images became scattered, from elliptical to quasi-rectangular to U-shaped. At high velocities, the profiles were curvy in the height dimension and even in the width dimension. It reveals that the vertical focusing quality decreased at high velocities while the horizontal focusing exhibited consistent high performance. In all cases, the observed dimensions kept below 23 *μ*m in both directions and the sample flow never deviated from the channel center more than a few micrometers. In the second experiment, the sample flow rate *Q_S_* was changed from 10 to 120 *μ*l/min and the sheath flow rate *Q_SH_* was fixed at 7.2 ml/min; therefore, the sample flow velocity *V_S_* was 4.8 m/s constantly. The results in Fig. 9 present that the focused sample flow had a quasi-square cross section. With the increasing sample flow rate, the height continuously increased from 13 to 22 *μ*m, and the area from 87 to 376 *μ*m^2^. The width changed from 8 to 21 *μ*m as the rate increased from 10 to 60 *μ*l/min, and then kept almost constant in the range of 60–120 *μ*l/min. The dimensions in the first experiment did not change as much as in the second experiment, since the ratio of *Q_SH_* to *Q_S_* was constant. These focusing performances presented by the chip were comparable to commercial instruments. For examples, BD Calibur works at 6 m/s and focuses the sample flow down to 6.5–15 *μ*m within the sample flow rate of 12–60 *μ*l/min, and BD Accuri C6 operates at 3 m/s with a focused diameter of 10–22 *μ*m and a sample flow rate of 14–66 *μ*l/min.
